# Muscle Mass and Vitamin B6 Are Linked to Negative Body Image in Women with Anorexia Nervosa: A Retrospective Study

**DOI:** 10.3390/nu16223902

**Published:** 2024-11-15

**Authors:** Federica Scarpina, Stefania Cattaldo, Elisa Prina, Paolo Piterà, Federico Brusa, Lorenzo Priano, Leonardo Mendolicchio, Alessandro Mauro

**Affiliations:** 1 “Rita Levi Montalcini” Department of Neurosciences, University of Turin, 10126 Turin, Italy; f.scarpina@auxologico.it (F.S.); e.prina@auxologico.it (E.P.); p.pitera@auxologico.it (P.P.); lorenzo.priano@unito.it (L.P.); alessandro.mauro@unito.it (A.M.); 2 I.R.C.C.S. Istituto Auxologico Italiano, U.O. di Neurologia e Neuroriabilitazione, Ospedale San Giuseppe, 282824 Piancavallo, Italy; 3 I.R.C.C.S. Istituto Auxologico Italiano, Laboratorio di Neurobiologia Clinica, Ospedale San Giuseppe, 282824 Piancavallo, Italy; 4 I.R.C.C.S. Istituto Auxologico Italiano, Laboratorio Sperimentale di Ricerche di Neuroscienze Metaboliche, Ospedale San Giuseppe, 282824 Piancavallo, Italy; f.brusa@auxologico.it (F.B.); l.mendolicchio@auxologico.it (L.M.); 5 I.R.C.C.S. Istituto Auxologico Italiano, U.O. di Riabilitazione dei Disturbi Alimentari e della Nutrizione, Ospedale San Giuseppe, 282824 Piancavallo, Italy

**Keywords:** anorexia nervosa, body image, physical body, body composition, vitamins

## Abstract

Introduction. Anorexia nervosa severely impacts the physical body and mental body (i.e., body image). In this retrospective study, we investigated the relationship between the perceived body image and body composition in women with anorexia nervosa. Specifically, we aimed to verify what components (i.e., weight, body composition, and micronutrients) may predict a higher number of symptoms of negative body image in this clinical condition. Methods. Weight status and body composition, including the expressions of vitamins, and body image concerns were measured in a sample of 112 women with anorexia nervosa (age in years M = 26.78; SD = 12; range = 14–67). Results. According to the regression analysis, a higher skeletal muscle mass and a higher concentration of vitamin B6 seemed to predict a higher number of symptoms of negative body image in our sample. Conclusions. This study pointed out muscle mass and the concentration of vitamin B6 as involved in the psychological expression of body image concerns in anorexia nervosa, especially at the beginning of the disease. Thus, we may suggest including and monitoring these parameters in routine care for anorexia nervosa.

## 1. Introduction

Negative body image is a core symptom of anorexia nervosa [1]. Affected individuals may show significant perceptual distortions in estimating physical body sizes (i.e., they tend to estimate their size as larger than is objectively true) and high levels of dissatisfaction toward bodily size, shape, and appearance [2,3,4]. The major expression of the disease symptoms is on the physical body: thinness and low weight (even if some people with anorexia nervosa may not look very thin) [5], blue fingers, thin and fragile hair, yellowing and dry skin, and downy hair covering the body [6,7]. Also, individuals with anorexia nervosa report high levels of weakness and tiredness; they feel dizziness or fainting [5]. Swelling of the arms and legs and stress fractures or reduced bone mass can be observed [8]. Some of these signs and symptoms mirror micronutrient deficiency, such as vitamin deficiency. In the context of eating disorders, vitamin D deficiency, together with an inadequate calcium intake, is highly associated with fractures, osteoporosis, and reduced bone mass [9]. Water-soluble vitamins like vitamin B complex and vitamin C are more prone to wash out from the body during stressful conditions, like starvation and low food intake [10]. Vitamins as micronutrients play a vital functional role in maintaining high levels of health and peak physical performance [11]. They enable those complex reactions necessary to use the potential energy in macronutrients to fuel the biological processes inherent in physical training and recovery [11,12]. Thus, in the case of reduced levels of vitamins, the physical body may be perceived as less strong and less capable, which is precisely the experience of individuals with anorexia nervosa. The lower level of strength mirrors what is observed in the clinical assessment of body composition in terms of reduction of fat mass, fat-free mass, and bone mass [13], and reduced efficiency of energy processes and proteolysis [14], as suggested by lower values of the phase angle (i.e., computed according to the resistance and reactance values obtained through bioelectric impedance analysis and directly correlated with body cell mass and cellular nutritional status). A lower phase angle reflects the impact of undernutrition, as observed in malnourished or cachectic patients [15]. Notably, phase angle values have been found to increase during nutritional recovery, along with improved body composition and fluid compartment changes [15,16]. Nevertheless, high inter-individual variability is observed in this parameter because of the role played by other factors, including individual levels of physical activity, the presence of vomiting and use of laxatives, the characteristics of the metabolism in starvation, and, possibly, on the stage of recovery.

In this retrospective study, we aimed to investigate the relationship between perceived body image and body composition (even at the level of micronutrients) in women with anorexia nervosa. Specifically, to recognize a sort of body-related biomarker of negative body image in anorexia nervosa, we verified what components (i.e., weight, body composition, and micronutrients) may predict a higher number of symptoms of negative body image in our sample.

## 2. Methods

### 2.1. Participants

This study was approved by the Ethics Committee of the involved institution (Reference number: 2022_09_27_10). All participants were volunteers who gave informed written consent. For individuals with ages lower than 18 years, parents signed the written consent. The Italian National Sanitary System covered all hospital charges, and participants were not remunerated.

In this retrospective observational study, only in-patients consecutively recruited at their admission to the hospital between February 2021 and March 2024 were included. Participants were evaluated according to routine procedures on a day-hospital basis. Individuals were included in this study if they satisfied the Diagnostic and Statistical Manual of Mental Disorders, Fifth Edition (American Psychiatric Association, 2013) criteria for anorexia nervosa, which are as follows: (i) restriction of food intake leading to weight loss or a failure to gain weight resulting in a “significantly low body weight” of what would be expected for someone’s age, sex, and height; (ii) fear of becoming fat or gaining weight; and (iii) a distorted view of themselves and their condition. Both “restricting type” and “binge eating/purging type” (American Psychiatric Association, 2013) were included in this study. Moreover, participants were included if they had not previously been treated, and none declared that they took prescribed or self-administered micronutrient supplements. Participants were excluded if other pathologies not related to anorexia nervosa were recorded (e.g., neurodegenerative diseases such as brain injury and stroke). Notably, individuals in their first recovery and those in their second or more recovery were included as the chronic nature of the disease [17], since affected individuals frequently undertake more than one treatment. A clinical assessment of the eating style through the traditional Eating Disorder Inventory™–3 (EDI-3) [18], which focuses on the symptomatology associated with eating disorders, and the Binge Eating Scale [19] to assess the presence of binge eating behavior. We administered the Symptom Checklist-90 [20], which allows the evaluation of self-reported severity of psychopathological symptoms, and the Italian version [21] of the Psychological General Well-Being Index (PGWBI) [22], which measures the self-reported level of anxiety, depressed mood, positive well-being, self-control, general health, and vitality.

### 2.2. Body Image

Our participants with anorexia nervosa filled out the Body Uneasiness Test [23], which assesses body image characteristics. This self-rating scale explores various areas of body-related psychopathology, which are dissatisfaction regarding the body and its weight; avoiding and compulsive control behavior; experience of separation and foreignness regarding the body; and specific worries for certain body parts, characteristics, or functions. The main global dimension of the Global Severity Index (i.e., GSI) is read as a marker of body image concerns, with a higher score indicating greater body uneasiness. This questionnaire has been validated in individuals with a body mass index lower than 25, showing a good internal consistency (Cronbach’s alpha coefficients range between 0.69 and 0.90 > 0.7) [23].

### 2.3. Weight Status and Body Composition

Because of the clinical standard routine, individuals’ weight and height were measured under standardized conditions in the morning, after a fasting period of 12 h, in light clothes without shoes. Body mass index (BMI) was calculated as body weight divided by squared height (kg/m^2^). Body weight (kg) and body height (meters) were measured with precision to the nearest 0.1 kg and 0.5 cm, respectively. A mechanical column scale (Scale-Tronix, Wheaton, IL) and a stadiometer (Scale-Tronix, Wheaton, IL, USA) were used for these measurements. Moreover, we performed a body composition analysis with a single-frequency bioelectrical impedance analysis (BIA 101, Akern^®^, Pisa, Italy).

After a 12 h fasting period, blood samples were collected through an indwelling cannula inserted at the cubital vein. Tubes were kept on ice and protected from light immediately after sampling. To determine the level of concentration of vitamin C, vitamin A, and vitamin E, plasma was separated with centrifugation at 3000× *g* for 10 min at 4 °C and stored at −80 °C until analysis. For vitamins B1 and B6, whole blood was stored at −80 °C until analysis. All vitamins were determined using high-performance liquid chromatography (HPLC) with commercially available kits from Chromsystems (Chromsystems Instruments and Chemicals GmbH, Gräfelfing, Germany), according to the manufacturer’s instructions. The inter- and intra-assay coefficients of variations were ≤4.2% and ≤2.3% for vitamin C, ≤3.5% and ≤4.9% for vitamin A, ≤3.2% and ≤4.1% for vitamin E, ≤5.9% and ≤4.5% for vitamin B1, and ≤5.2% and ≤4.0% for vitamin B6.

### 2.4. Statistical Analysis

Data were initially analyzed using descriptive statistics, including means, standard deviations, frequencies, and percentages. Scores about the psychological questionnaires were computed according to the seminal articles.

We performed preliminary analyses to describe our sample of participants with anorexia nervosa in terms of clinical parameters. First, we considered the value of body mass index. We split the sample into two groups: participants with a score below the threshold of 18 (i.e., underweight) and those over this threshold (i.e., normal weight). Through an independent sample *t*-test, we verified any difference between the two groups in the score of the Global Severity Index. We followed the same rationale about the different parameters from the bioelectrical impedance analysis. However, to our knowledge, there is no specific threshold for these parameters in the literature about anorexia nervosa. Thus, we split the sample into two groups, assessing if each individual’s score was below or over the within-sample median. Through an independent sample *t*-test, we verified any difference between the two groups in the score of the Global Severity Index. Concerning vitamins, we verified how many participants reported a concentration within, below, or over the normal range. The reference levels of vitamins were obtained from the datasheets of the kits used for their analysis. Thus, according to the one-way analysis of variance, we verified any difference between the three groups at the score of the main index of the Global Severity Index.

Successively, we verified any relationship between the role of age, age at the onset, and disease length, and the parameters relative to the body image and the physical body.

Finally, we used a linear regression analysis approach to investigate whether and what body image concerns (the outcome, the Global Severity Index) may be associated with weight status and body composition (the predictive factors). Indeed, this statistical model is used to quantify the strength of the relationship between the outcome and the explanatory variables. First, the correlation and directionality of the data were investigated in a preliminary analysis to formulate the statistical model using Pearson’s correlation coefficient (Pearson’s r). Those variables significantly associated (*p*-value 0.05) with the score relative to the main outcome were further investigated with a multiple linear regression model. Goodness-of-fit was reported as R^2^; the significance of the model was evaluated according to the F-value and *p*-value. The relative contribution of factors included in the statistical model was verified with the independent variable. Finally, for each predictive factor included in the model, the variance inflation factor (VIF) was reported as a measure of multicollinearity.

## 3. Results

### 3.1. Participants

Data relative to 112 women with anorexia nervosa were collected (age in years M = 26.78; SD = 12; range = 14–67; age in years at the symptoms onset M = 18.02; SD = 6.36; range = 10–51; disease length in years M = 8.77; SD = 10.35; range: 1–47). Overall, the majority of the sample (92.85% N = 104) satisfied the criteria for the restrictive type, while 7.14% (n = 8) of the sample was categorized as a binge/purging type. The majority (N = 63; 56.25%) of our sample was in early adulthood (18 years old to mid-30s), while 19.64% (N = 22) were adolescents (age ≤ 18 years old). Moreover, 22.32% (N = 25) was in adulthood (mid-30s to mid-60s), and only 1.78% (N = 2) was in late adulthood (mid-60s). In Table 1, the results relative to the psychopathological assessment of our participants are reported.

### 3.2. Body Image

The results of the Body Uneasiness Test [23], which measures body image, are reported in Table 2.

### 3.3. Weight Status and Body Composition

Table 3 reports the results for the parameters used to describe the physical body.

We did not find any difference in the score at the Global Severity Index between participants with a score below the threshold of 18 in body mass index (i.e., underweight; N = 98; M = 3.16; SD = 1.17) and those over the threshold (i.e., normal weight; N = 14; M = 3.61; SD = 0.79) [t(110) = 1.41; *p* = 0.15]. When the following rationale was used about the parameters relative to the bioelectrical impedance analysis, we observed a significant difference in the Global Severity Index when the sample was split according to the phase angle, body cell mass, fat mass, body cell mass index, skeletal muscle mass, and fat-free mass index, as reported in Table 4.

According to the results reported in Table 5, we did not find any significant differences in the score at the Global Severity Index when our sample was split into three groups according to the expression of the vitamins (i.e., within, below, or over the normal range). The only exception was the results relative to vitamin C: participants with a concentration below the normal range reported a significantly lower score at the Global Severity Index than those within the normal range.

### 3.4. The Role of Age, Age at the Onset, and Disease Length

As shown in Table 6, more negative symptoms in almost all the components measured by the questionnaire relative to body image were significantly related to lower demographic age, age at the onset, and disease length. The only exception was the Positive Symptom Distress Index (PSDI) (i.e., the mean intensity of all disliked body parts), which was not significantly related to the demographic parameters. As observed in Table 7, older participants reported significantly a lower fat mass and a lower skeletal muscle mass, as well as lower concentrations of vitamin B6 but higher levels of extracellular water. A longer disease length was associated with lower fat mass, lower skeletal muscle mass, and lower expressions of vitamin B6. As shown in Table 8, when we considered the physical parameters of body mass index and body impedance analysis, we observed a significant relationship between multiple scores relative to the body image concerns. The sign of the coefficient was positive, meaning that a more muscular body (as suggested by higher scores for body mass index, phase angle, fat-free mass, body cell mass, fat mass, muscle mass, body cell mass index, skeletal muscle mass, and fat-free mass index) was significantly related with a more negative body image (as suggested by higher scores at the psychological questionnaire). The only exceptions were represented by the parameters relative to the parameters of extracellular water and hydration, about which we observed a negative sign of correlation. About vitamins, we observed a less pronounced pattern. The most consistent result emerged about vitamin B6: a higher expression of this vitamin was significantly associated with a higher Global Severity Index of negative body image. Moreover, it was related to higher expressions of avoidance-related behaviors and a higher score at the positive symptom total (PST) relative to body uneasiness. Finally, higher expressions of vitamin C were related to higher positive symptom total (PST) of body uneasiness, and lower expressions of vitamin E were related to more severe body image concerns (BIC).

### 3.5. Regression Analysis

The Global Severity Index was included in the model as the main outcome. We introduced the parameters of body mass index (VIF = 4.32), phase angle (VIF = 5.632), extracellular water (VIF = 7.99), hydration (VIF = 6.07), skeletal muscle mass (VIF = 1.64), fat-free mass index (VIF = 4.56), and B6 (VIF = 1.02) as predictors since they were significantly related to the main outcome (Table 6). Notably, other parameters relative to bioelectrical impedance analysis emerged as significantly correlated to the main outcome. However, they showed a very high multicollinearity. Then, we selected those parameters that may be globally descriptive in the case of anorexia nervosa [14,15,24]. The model was significant [R2 = 0.237; F(7,95) = 3.903; *p* = 0.001]. Skeletal muscle mass [B = 0.145; *p* = 0.008] and B6 [B = 0.01; *p* = 0.026] were significant; no other parameters were significant [*p* ≥ 0.17]. Thus, higher levels of skeletal muscle mass and higher concentrations of vitamin B6 significantly predicted a higher level of body image concerns in our sample.

## 4. Discussion

Anorexia nervosa severely impacts both the physical and mental body. In this retrospective study, we aimed to identify any specific component of the physical body (even in the micronutrients) affecting the mental body. According to our results, two physical body-related components were significantly related to a negative body image: the skeletal muscle mass and the concentration of vitamin B6.

It is well known that in anorexia nervosa, we generally observe a loss of body fat. Interestingly, skeletal muscle mass is also often reduced [25], with negative side effects on the levels of vitamin D, bone mineral density, bodily strength, metabolic function, and physical performance [26]. Overall, individuals with anorexia nervosa have smaller muscle size and reduced energy expenditure when compared with healthy controls. Additionally, some studies reported that the recovery from this clinical disease was not enough to restore muscle mass. It should be observed that a muscular body, especially in women, is far from the ideal mental body in anorexia nervosa, while it is more characteristic of orthorexia [27], which is an eating disorder in which people express an obsessive focus on healthy eating, and bigorexia [28,29], which is a body dysmorphic disorder in which individuals express the desire to have less fat mass and the obsession with increasing muscle mass. Interestingly, our results suggest that a lower skeletal muscle mass is associated with older age and longer disease length, in line with the evidence that this parameter generally does not restore in the long term. Moreover, younger women at the beginning of anorexic symptoms could still have a muscular body, which may, in turn, increase the negative body image. Thus, our evidence that a higher skeletal muscle mass predicts more negative body image concerns is not surprising.

The novelty of this research is the result relative to the micronutrient component of vitamin B6. Our results suggested that it may play a role in body image in anorexia nervosa. We observed that a higher concentration of vitamin B6 was significantly related to higher negative body image. Vitamin B6 is water soluble and can be obtained from different foods and supplements. In the healthy population, its deficiency is not very common [30,31]; instead, its lower expression generally occurs in eating disorders, including anorexia nervosa, due to insufficient dietary intake, malabsorption, and use of certain medications [32,33]. Malnourished, elderly, and anorexic individuals are at higher risk of developing vitamin B6 deficiency [32,34], with several side effects such as anemia, peripheral neuropathy, seborrheic dermatitis, glossitis, cheilosis, depression, celiac disease, and seizures [35,36]. Our data seems to support this evidence since, in our sample of participants with anorexia nervosa, lower concentrations of vitamin B6 were significantly associated with older age and longer disease length. However, how do we conceive the relationship between the concentration of vitamin B6 and a negative body image? To our knowledge, this relationship was not explored in the literature. B vitamins directly impact bodily energy levels, brain function, and cell metabolism. Thus, when their expression is within the normal range, people perceive the physical body as healthy and energized. However, in individuals with anorexia nervosa, an energized body may be miscategorized as a fat body and mislabeled as the experience of “feeling fat” [37], enhancing negative thoughts, emotions, and feelings (i.e., body image). On the other hand, the decreasing levels of B vitamins, which may result in perceiving the physical body as less strong, may decrease the expressions of body image concerns. Because of the lack of evidence in the literature, our hypothesis about the predictive role of the expression of vitamin B6 on body image concerns is speculative, and we cannot further establish the mechanism underlying this association. We need future investigation to confirm our hypothesis. For example, experimental studies exploring the physiological and psychological effects of vitamin B6 supplementation in individuals with anorexia nervosa could further clarify the role of this micronutrient in body image concerns.

We underlined some limitations of this study. Our sample consisted only of female participants, since there is a higher incidence of anorexia nervosa in women than in men [38]. Moreover, it is well-established that bodily perceptions and body images are different between genders [39,40,41]. Consequently, the evidence from this research should not be generalized to males. Moreover, it was a monocentric-based study, limiting the generalizability of our findings. It should be noted that the sample’s age ranged from 14 to 67 years old, meaning that different phases of life (from adolescence to elderly) were included in the sample. In future cross-sectional studies, the age of the participants at the time of the experimental observation should be used as an inclusion/exclusion criterion. We furnished a clinical description of the global psychological functioning of our sample as well as the characteristics of the eating disorder and the expression of psychopathological symptoms. However, because of the sample size, we did not include any of this information in the statistical model (otherwise, the statistical power would be dramatically decreased). Thus, in the case of larger samples, we may suggest verifying the role of demographical information and disease-related characteristics (including the level of severity) on the main outcome. Finally, we used a self-report measurement that is highly used in clinical and research contexts to assess the mental body. However, body perceptions, especially about physical dimensions, could be measured using behavioral instruments, which should be included in future research.

## 5. Conclusions

Our evidence may suggest the level of muscle mass and the concentration of vitamin B6 as key components in experiencing a negative body image in anorexia nervosa, especially at the beginning of the disease. Notably, the role of muscle mass has some evidence in the literature, while the role of vitamin B6 in this context represents a novel and intriguing result. Because of this observation, future research is needed to support this novel evidence. Nevertheless, we strongly suggest the monitoring of these components in the routine care for anorexia nervosa, especially before and after multidisciplinary treatments.

## Figures and Tables

**Table 1:** Mean, standard deviation, and range relative to the scores at the questionnaires on eating behavior (Binge Eating Scale and Eating Disorders Inventory-3), symptoms of psychopathology (Symptom Checklist-90), and overall psychological well-being (Psychological General Well-Being Index) in our sample of participants affected by anorexia nervosa.

	Mean	Standard Deviation	Min	Max
**Binge Eating Scale**				
Score	31.27	10.12	10	102
**Eating Disorder Inventory™–3**				
Drive for thinness	22.10	8.27	0	28
Bulimia	6.21	7.32	0	32
Body dissatisfaction	29.04	8.61	2	40
Eating disorder risk	57.35	18.20	11	96
Low self-esteem	16.04	5.98	1	24
Personal alienation	15.31	6.44	2	28
Interpersonal insecurity	12.79	6.52	0	26
Interpersonal alienation	12.19	5.68	1	28
Interoceptive deficits	22.58	16.87	0	171
Emotional dysregulation	10.93	7.13	0	37
Perfectionism	11.38	5.42	11	24
Asceticism	14.54	6.44	1	28
Maturity fears	17.59	7.78	0	32
Ineffectiveness	31.36	11.81	5	52
Interpersonal problems	24.98	11.19	3	51
Affective problems	32.04	14.52	0	63
Overcontrol	25.92	10.06	2	51
General psychological maladjustment	131.89	43.58	26	237
**Symptom Checklist-90**				
Somatization	1.94	1.02	0	6.17
Obsession-compulsion	2.28	0.84	0	3.90
Interpersonal sensitivity	2.23	0.93	0.11	3.89
Depression	2.29	1	0	6.85
Anxiety	2.29	0.99	0.1	7.20
Hostility	1.28	0.97	0	3.67
Phobic anxiety	1.34	0.90	0	3.43
Paranoid ideation	1.9	0.83	0	3.67
Psychoticism	1.42	0.66	0	3.4
Total score	2.27	2.86	0.13	31.19
**Psychological General Well-Being Index**				
Anxiety	8.31	5.57	0	24
Depression	5.6	4.27	0	14
Positive well-being	4.63	3.38	4	15
Self-control	6.22	3.23	0	14
General healthy	7.20	3.09	0	14
Vitality	7.02	4.27	0	19
Total score	38.98	18.14	4	92

**Table 2:** Mean, standard deviation, and range relative to the scores at the questionnaire relative to body image (Body Uneasiness Test [23]) in our sample of women affected by anorexia nervosa.

	Mean	Standard Deviation	Min	Max
Global Score Index	3.22	1.11	0.5	6.09
Weight phobia	3.64	1.19	0.25	5
Body image concerns	3.59	1.15	0.78	5
Avoidance	2.57	1.51	0	12.83
Compulsive self-monitoring	3.08	1.39	0	5
Depersonalization	2.88	1.36	0.2	6.4
Positive symptom total	22.14	9.8	1	37
Positive Symptom Distress Index	3.23	0.84	1.36	5.67

**Table 3:** Mean, standard deviation, and range relative to the scores describing the physical body in our sample of women with anorexia nervosa.

	Mean	Standard Deviation	Min	Max
Body Mass Index	15.01	2.41	9.74	20.89
**Body Impedance Analysis**				
Phase angle (°)	3.88	1.11	1.03	6.06
Fat-free mass (%)	34.04	4.86	15.30	45.4
Body cell mass (%)	14.77	4.37	1.4	28
Fat mass (%)	13.33	7.52	4.8	29.7
Extracellular water (%)	56.17	8.6	38.4	86.7
Muscle mass (%)	47.54	7.67	18.5	68.8
Body cell mass index	5.55	1.52	0.7	9.4
Hydration (%)	73.78	4.36	62.7	90
Skeletal muscle mass (kg)	17.38	2.5	8.1	24.1
Fat-free mass index	12.85	1.35	9.20	16.1
**Vitamins**				
Vitamin C (mg/L)	12.03	5.96	0.20	30.80
Vitamin A (mg/L)	0.51	0.21	0.11	1.15
Vitamin E (mg/L)	15.8	5.49	2.84	37.4
Vitamin B1 (μg/L)	57.61	18.7	23.8	129
Vitamin B6 (μg/L)	31.24	17.42	6.4	109.58

**Table 4:** For each parameter of the bioelectrical impedance analysis, sample size, mean, and standard deviation relative to the Global Severity Index from the psychological questionnaire were reported when the sample was split into two groups according to the within-sample threshold. In bold, significant results (*p* < 0.05).

	Within-Sample Threshold	Below the Threshold	Over the Threshold	Statistical Results
Phase angle (°)	4.035	n = 61 M = 3 SD = 1.18	n = 51 M = 3.48 SD = 0.94	t(110) = 2.35; ***p* = 0.02**
Fat-free mass (%)	34.5	n = 60 M = 3.07 SD = 1.10	n = 52 M = 3.39 SD = 1.09	t(110) = 1.55; *p* = 0.12
Body cell mass (%)	14.7	n = 59 M = 2.94 SD = 1.13	n = 53 M = 3.53 SD = 0.99	t(110) = 2.9; ***p* = 0.004**
Fat mass (%)	10.9	n = 60 M = 3.01 SD = 1.1	n = 52 M = 3.46 SD = 1.07	t(110) = 2.18; ***p* = 0.03**
Extracellular water (%)	54.3	n = 64 M = 3.35 SD = 0.99	n = 48 M = 3.04 SD = 1.23	t(119) = 1.48; *p* = 0.15
Muscle mass (%)	48	n = 63 M = 3.06 SD = 1.2	n = 49 M = 3.42 SD = 0.93	t(110) = 1.71; *p* = 0.08
Body cell mass index	5.75	n = 61 M = 2.92 SD = 1.16	n = 51 M = 3.57 SD = 0.93	t(110) = 3.23; ***p* = 0.002**
Hydration (%)	73.2	n = 57 M = 3.23 SD = 1.01	n = 55 M = 3.11 SD = 1.19	t(110) = 0.99; *p* = 0.32
Skeletal muscle mass (kg)	17.7	n = 57 M = 2.93 SD = 1.11	n = 55 M = 3.52 SD = 1.02	t(110) = 2.92; ***p* = 0.004**
Fat-free mass index	12.85	n = 61 M = 2.93 SD = 1.16	n = 51 M = 3.57 SD = 0.92	t(110) = 3.16; ***p* = 0.002**

**Table 5:** Sample size, mean, standard deviation, and range (minimum and maximum) relative to the Global Severity Index from the psychological questionnaires are reported for the sample when it was divided into three groups about the level of concentration of the vitamins (i.e., within, below or over the normal range). In bold, significant results (*p* < 0.05).

					Range		
	N	Mean	Standard Deviation		Min	Max	Statistical Results
**Vitamin C**							
Within the normal range	68	3.35	1.11	0.14	0.68	6.09	F(2,111) = 3.54; *p* = 0.032 normal vs. below *p* = 0.02; other comparison ≥ 0.08
Below the normal range	13	2.48	1.21	0.34	0.5	4.62	
Over the normal range	31	3.27	0.95	0.17	0.94	4.5	
**Vitamin A**							
Within the normal range	72	3.35	1.11	0.13	0.68	6.09	F(2,111) = 1.44; *p* = 0.23
Below the normal range	18	2.95	1.17	0.28	0.5	4.85	
Over the normal range	22	3.02	1.01	0.22	1.09	4.5	
**Vitamin E**							
Within the normal range	90	3.25	1.07	0.11	0.5	4.85	F(2,111) = 0.58; *p* = 0.55
Below the normal range	1	2.12			2.12	2.12	
Over the normal range	21	3.14	1.28	0.28	0.68	6.09	
**Vitamin B1**							
Within the normal range	102	3.22	1.12	0.11	0.5	6.09	F(2,111) = 0.12; *p* = 0.88
Below the normal range	2	3.58	0.19	0.14	3.44	3.71	
Over the normal range	8	3.14	1.12	0.39	1.09	4.12	
**Vitamin B6**							
Within the normal range	59	3.07	1.15	0.15	0.5	4.85	F(2,111) = 1.36; *p* = 0.26
Below the normal range	1	2.82	–	–	2.82	2.82	
Over the normal range	52	3.41	1.05	0.15	0.85	6.09	

**Table 6:** Pairwise correlation matrix among the scores reported at the questionnaire relative to body image (columns) and the parameters of demographical age, age at the onset, and disease length. In bold, *p* ≤ 0.05; in bold and italic, *p* ≤ 0.001.

	Age	Age at the Onset	Disease Lenght
GSI	***−0.580***	***−0.374***	***−0.441***
WP	***−0.466***	***−0.263***	***−0.378***
BIC	***−0.580***	***−0.415***	***−0.417***
Av	***−0.436***	***−0.314***	***−0.311***
CSM	***−0.519***	***−0.280***	***−0.428***
D	***−0.453***	***−0.283***	***−0.351***
PST	***−0.528***	***−0.431***	***−0.347***
PSDI	−0.142	−0.158	−0.067
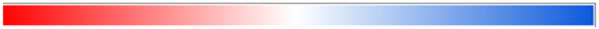			
**−1** **+1**			

**Table 7:** Pairwise correlation matrix among the scores reported at the parameters relative to the physical body (columns) and the parameters of demographical age, age at the onset, and disease length. In bold, *p* ≤ 0.05; in bold and italic, *p* ≤ 0.001.

	Age	Age at the Onset	Disease Lenght
Body Mass Index	−0.139	0.029	−0.180
Phase Angle	−0.129	−0.147	−0.059
Fat-Free Mass	−0.125	−0.028	−0.127
Body Cell Mass	−0.174	−0.098	−0.142
Fat Mass	***−0.245***	−0.111	**−0.216**
Extra Cellular Water	***0.209***	0.137	0.155
Muscle Mass	−0.122	−0.092	−0.083
Body Cell Mass Index	−0.153	−0.099	−0.116
Hydration	0.122	0.149	0.048
Skeletal Muscle Mass	***−0.391***	−0.100	***−0.384***
Fat-Free Mass Index	−0.033	0.017	−0.049
Vitamin C	−0.135	−0.160	−0.057
Vitamin A	0.061	0.158	−0.026
Vitamin E	0.166	0.185	0.078
Vitamin B1	0.053	0.125	−0.015
Vitamin B6	***−0.246***	−0.158	**−0.188**
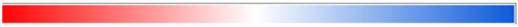			
**−1** **+1**			

**Table 8:** Pairwise correlation matrix among the scores reported at questionnaire relative to body image (columns) and the parameters describing the physical body (i.e., body mass index and the parameters relative to body impedance analysis) and its micronutrients (i.e., vitamins). In bold, *p* ≤ 0.05; in bold and italic, *p* ≤ 0.001.

	Body Mass Index	Phase Angle	Fat-Free Mass	Body Cell Mass	Fat Mass	Extra Cellular Water	Muscle Mass	Body Cell Mass Index	Hydration	Skeletal Muscle Mass	Fat-Free Mass Index	Vitamin C	Vitamin A	Vitamin E	Vitamin B1	Vitamin B6
GSI	**0.250**	***0.305***	**0.241**	***0.309***	0.172	***−0.303***	**0.239**	***0.312***	−0.178	***0.310***	**0.227**	0.136	−0.038	−0.138	−0.031	**0.191**
WP	***0.348***	***0.371***	***0.311***	***0.384***	**0.244**	***−0.390***	***0.298***	***0.397***	**−0.252**	***0.275***	***0.320***	0.101	0.029	−0.122	−0.010	0.118
BIC	**0.244**	***0.325***	**0.241**	***0.332***	0.173	***−0.309***	**0.241**	***0.329***	**−0.201**	***0.314***	**0.217**	0.155	−0.118	**−0.212**	0.002	0.176
Av	0.038	0.080	0.053	0.080	0.017	−0.081	0.080	0.079	−0.012	**0.203**	0.037	0.056	−0.044	−0.037	−0.076	**0.214**
CSM	***0.283***	***0.272***	**0.253**	***0.279***	**0.222**	***−0.268***	0.176	***0.273***	−0.145	***0.287***	**0.234**	0.177	−0.055	−0.105	−0.044	0.163
D	0.147	**0.240**	**0.205**	***0.289***	0.045	***−0.284***	***0.282***	***0.288***	−0.163	***0.274***	0.183	0.064	0.115	−0.106	−0.001	0.130
PST	**0.203**	0.188	0.113	0.139	**0.225**	−0.183	0.103	0.166	−0.137	***0.264***	0.109	**0.213**	−0.003	0.089	0.045	**0.186**
PSDI	***0.269***	***0.362***	0.176	***0.322***	**0.205**	***−0.322***	**0.204**	***0.332***	***−0.284***	−0.062	**0.218**	−0.057	0.013	−0.010	−0.065	−0.056
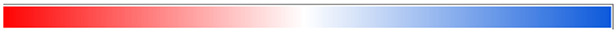																
**−1** **+1**																

## Data Availability

The data that support the findings of this study are available in Zenodo at the following link: https://doi.org/10.5281/zenodo.13923377.
